# Continuance intentions to use FinTech peer-to-peer payments apps in India

**DOI:** 10.1016/j.heliyon.2022.e11654

**Published:** 2022-11-14

**Authors:** Basri Savitha, Iqbal Thonse Hawaldar, Naveen Kumar K

**Affiliations:** a Manipal Institute of Management, Centre for Advanced Research in Financial Inclusion, Manipal Academy of Higher Education, Manipal 576104, Karnataka, India; b Department of Accounting & Finance, College of Business Administration, Director, Accreditation & Quality Assurance Centre, Kingdom University, Bahrain; c National Institute of Bank Management, Kondhwe Khurd, Pune 411048, India

**Keywords:** FinTech, Continuance intention, Person-to-person, Confirmation, Co-creation, Trust, Social influence, Extended expectancy confirmation model

## Abstract

**Purpose:**

The purpose of the present study is to identify the determinants of continuance intentions to use FinTech peer-to-peer (P2P) payment apps in India.

**Design:**

A cross-sectional survey of 463 FinTech users was carried out during the pandemic with the help of a digital questionnaire. The study has empirically tested an extended expectancy confirmation model and theory of acceptance to examine the determinants of continuance intentions to use FinTech P2P payment apps.

**Findings:**

We demonstrate that confirmation of prior expectations and post-adoption perceived usefulness determine continuance intention to use FinTech payment services. Cognitive acceptance (trust) and normative acceptance (social influence) not only determine behavioural acceptance (willingness to co-create) but also influences continuance intentions. Confirmation of initial expectations during service use determines satisfaction and post-purchase perceived usefulness.

**Practical implications:**

The continuous use of FinTech P2P services can be ensured by fulfilling users' post-purchase expected benefits, fostering trust, and social influence. These can be achieved by gearing up internal resources to provide customized experiences that meet customers’ expectations and facilitate fruitful interactions. Only if the mobile experience is accessible and meets the expectations of customers, they would appreciate its performance and value in the offerings leading to extensive use of FinTech P2P services.

**Originality/value:**

The present paper is based on extended ECM and the theory of acceptance that aim to explain continuance intention to use FinTech P2P payments. The study findings add to the limited body of research in the context of FinTech P2P payments.

## 1 Introduction

The accelerated growth in digital transactions witnessed during the coronavirus pandemic in India can be attributed to the FinTech industry that has provided online payment platforms for seamless transactions. The pandemic has propelled the growth of digital payments markets, which has been estimated to increase from USD 2.33 trillion (I USD = INR 78.01, as of 15 June 2022) in the year 2021 to USD 58.95 trillion by the year 2024 and would grow at a compound annual growth rate of 22% during the next five years (KPMG, 2020; NPCI, 2020). Digital transactions declined by 49% in the initial months of lockdown, but conditional relaxations in the second half of the year 2020 fastened the rate of adoption of digital over cash payments (NPCI, 2020). The COVID-19 lockdown effect was seen in negative growth of payments in March and April 2020 (−17%), but the relaxations in May 2020 resulted in a 44% gain in payments, which thereafter dropped to an average of 11.18% during which a few cases of COVID-19 infections were documented (June to August 2020), and a growth of 10.3% was witnessed during the first wave of the COVID-19 (September 2020). Following that, there was a drop in growth rate (−2.4% during November 2020 and February 2021) until the second wave hit India in March 2021 (18.78% gain) (RBI, 2021) and then a negative growth was seen in the initial months (an average of −1.42% in April and May 2021) after the waning of the second wave (RBI, 2021). Based on these reflections, we can expect a gradual decline in incremental growth if the pandemic recedes. According to NPCI (2020), cash is the preferred way of payment in the informal sector of India's semi-urban and rural areas, where the structure of the payment network is less developed. Based on a multi-country study, Gene and Chakravorti (2009) conclude that cash usage continues to dominate despite the increased rise in digital payments in OECD countries. India, too, offers an intriguing scenario in which both cash and cashless payments coexist, as well as a transformation in the payments ecosystem. As a result, adopters of FinTech during the COVID-19 pandemic may switch to cash payments after the pandemic if FinTech firms do not meet their requirements and engage customers effectively.

In this scenario, customer retention and continuing use of digital payments even after a pandemic necessitate an understanding of the relational nature of FinTech services, in particular, the role of acceptance of firms' value proposition and confirmation of expectation and trust is essential. It is a well-known fact that gaining a new client costs more than keeping an existing one, and businesses that are unable to ensure that their services are used regularly will lose clients (Zhou, 2013). If the service experience does not meet users’ expectations, they would cease utilizing it, resulting in discontent or distrust (Nguyen and Simkin, 2017; Chen and Li, 2017; Chuah et al., 2017). Therefore, continual use of services is required to recover expenses and preserve the long-term viability of businesses (Bhattacherjee, 2001a; Yuan et al., 2016; Bhattacherjee and Premkumar, 2004). As a result, FinTech service providers grapple with the challenge of enticing customers to accept their value proposition and maintain early adopters' enthusiasm by fulfilling promises. Therefore, FinTech product development and marketing teams should concentrate on what matters to customers in terms of embracing the value proposition and how effectively can they provide superior value to retain the customer base.

The literature on the continuation of services in virtual payment platforms especially peer-to-peer payments (P2P) is scanty. Studies on continuance intention carried out in different contexts conclude that factors influencing initial adoption and subsequent post-consumption phase are varied due to changes in consumer perception after the initial user experience (Venkatesh et al., 2011; Bhattacherjee, 2001a,b; Bhattacherjee and Premkumar, 2004). Therefore, the present paper tries to contribute to the literature on the influence of cognitive, affective, and normative acceptance of value offering and service co-creation intention (behavioural acceptance) and expectancy-confirmation-satisfaction relationship on continuous usage intentions in new FinTech technological settings. The findings would be useful for the FinTech companies and other stakeholders wanting to retain the existing FinTech P2P payment user base in India.

The Expectation-Confirmation Model (ECM) proposes that continuance intention (CI) to use information systems depends on initial usage experience (satisfaction) and expectations of future benefits (post-usage usefulness), which are in turn determined by confirmation of prior expectations of service usage (Bhattacherjee, 2001a,b). However, when applying ECM to consumer behaviour, the necessity of acceptability of the company's value proposition should be considered. Customers are more likely to continue to use services if they accept the value proposition. The promise of a value proposition that solves the most severe pains and consistently provides large gains is crucial to client retention. The research on the influence of acceptance of value on continuance intention to use P2P payments is limited, despite the emerging premise that it matters the most in customer decisions in interdependent technology-intensive FinTech services. Therefore, the present paper extends the ECM framework (Bhattacherjee, 2001a; Bhattacherjee and Premkumar, 2004; Oghuma et al., 2015a,b; Gong et al., 2018) by integrating it with the theory of acceptance that includes cognitive acceptance (trust in service providers), behavioural acceptance (willingly participate in co-creating services), and normative acceptance (social influence) (Tax et al., 1998; Bearden et al., 1989; Shulga and Busser, 2020) in explaining continuance intention to use P2P FinTech payment apps.

## 2 Literature review

Several researchers (Gong et al., 2018; Kumar et al., 2018; Liao et al., 2009; Hoehle et al., 2012) on the continued use of a system that regards customer satisfaction and post-usage usefulness (PU) being decided by confirmation of expectation have employed Oliver's (1980) initial expectation-disconfirmation theory and Bhattacharjee's (2001a) modified expectation-confirmation model (ECM). The ECM ascribes continuance intention as a function of satisfaction with the initial use of services and their expected usefulness in future consumption. Since technology-intensive services offered in a virtual environment change with time when new features or capabilities are added to later versions, initial expectations as a predictor of continuance intention are inappropriate (Bhattacherjee and Barfar, 2011). Prior expectations can change in subsequent use when consumers lower their unreasonable high level of initial expectation after the disconfirming actual user experience. There are possibilities of users increasing their expectations from low prior expectations after positive confirmation during the post-adoption phase (Bhattacherjee, 2001a,b).

The theory of acceptance, which emphasizes the importance of value proposition acceptance on consumers' desire to participate in value co-creation and continuing use of services, might also explain the continuity intention. The theory proposes that endorsement of firms' value proposition depends on behavioural, cognitive, and normative reasoning, as well as a sincere and purposeful affirmation (Cohen, 1992; Steel, 2013; Maher, 1993; Shulga and Busser, 2020). For example, a FinTech services customer may cognitively reject the firm offerings as risky and untrustworthy, but he or she may nevertheless accept the service after seeing their friends or family members use it. As a result, a normative or cognitive value judgment affects user behaviour. Cognitive acceptance is defined as a trust in propositions based on truth, validity, and factualness that influences one's desire to maintain a long-term preference and relationship with service providers (Audi, 2008; Gounaris, 2005; Shulga and Busser, 2020). Users would develop a long-term relationship with the company by cooperating and engaging in co-creating products or services if they trust the security and confidentiality of information promised by the company (Cazier et al., 2007; Kosiba et al., 2018; Chai and Kim, 2010).

According to normative acceptance, identification, internalization, and compliance due to interpersonal influence are reflected in compliance with preferences and conformity to the expectations of significant others and the social environment (Bearden et al., 1989; Forgas and Williams, 2001; Sunshine and Tylor, 2003; Shulga and Busser, 2020). The term ‘social influence’ is frequently used to describe a normative and internalized obligation. Behavioural acceptance is measured as the willingness to participate in co-creation or the desire to spread positive or negative perceptions of value offering through word-of-mouth (Zeithaml et al., 1996; Bolton and Saxena-Iyer, 2009). ‘Value-in-use’ service-dominant logic stresses that consumers purchase products or services to create value in the form of knowledge or skills gained from its use (Vargo and Lusch, 2004). Also, users expect to interact and creatively engage with companies right through product improvement and purchase process and communicate and share their experiences to create value for mutual benefits (Payne et al., 2008; Prahalad and Ramaswamy, 2000). The literature on the constructs of the study is discussed below.

### 2.1 Confirmation

During the service experience, initial assumptions influenced by advertising, digital marketing campaigns, information search, and feedback from other users are either verified or disproved. If a customer's transactional experience meets or surpasses his or her expectations, the user will consider the transaction to be worth the money and effort, and hence will be satisfied with the services. Users who are satisfied plan to continue using the service, whilst those who are unsatisfied want to stop using it. Consumers form a psychological or emotive judgment of previous use based on a comparison of the cognitive assessment of pre-adoption and post-acceptance expectation matching, resulting in a positive or negative opinion (Bhattacherjee, 2001a,b) and thereby, decide to continue or discontinue the use of services (Oghuma et al., 2016). Therefore, confirmation (CF) would affect satisfaction (Zhou et al., 2018; Hoehle et al., 2012; Bhattacherjee, 2001a). Furthermore, disconfirmation or cognitive dissonance affects post-adoption usefulness (PU). To mitigate the negative effects of dissonance, consumers might change their perceptions or behaviour. Despite their uncertainties about a product's or service's perceived utility, consumers may accept it at first and decide whether or not to use it again based on their post-adoption experience and changed perspectives. The PU will be higher if the user experience confirms the expectations (Mou et al., 2017; Sarkar and Khare, 2018; Tam et al., 2018; Khayer and Bao, 2019). Few studies have concluded a positive effect of confirmation on post-usage usefulness (Oghuma et al., 2016; Bhattacherjee, 2001a,b; Foroughi et al., 2019; Hoehle et al., 2012, Liao et al., 2009; Zhou et al., 2018; Susanto et al., 2016). Thus, we hypothesize that confirmation influences post-usage usefulness (H1) and satisfaction (H2).

### 2.2 Satisfaction

A service provider's success is determined by their ability to deliver promised value and anticipate and effectively manage client expectations. Satisfaction (ST) depends on positive confirmation resulting from post-purchase performance matching or exceeding pre-purchase expectations (Oliver, 1993). When the prior expectation is fulfilled during the service experience, the consequent positive confirmation leads to higher satisfaction. In the same way, higher expectations and lower performance give rise to disconfirmation and the subsequent dissatisfaction ensues discontinuance intention (Oghuma et al., 2016; Venkatesh et al., 2011; Premkumar, 2004; Bhattacherjee, 2001a,b). Thus, confirmation influences satisfaction which in turn shapes continuance intention to use. Satisfaction from past usage primarily influences continuance intention (Bhattacherjee, 2001a; Tran et al., 2019; Ofori et al., 2017; Khayer and Bao, 2019; Chen et al., 2009; Rahi et al., 2021; Puriwat and Tripopsakul, 2021; Susanto et al., 2016). Hence, we propose that satisfaction with the service positively influences continuance intention to use P2P payments (H3).

### 2.3 Post-usage usefulness

ECM incorporates subtleties of user-related beliefs in technology usage intentions by stressing the role of PU determined by expectations in the post-acceptance phase and confirmation of expectations from prior use performance (Bhattacherjee, 2001a,b). Post-purchase perceived usefulness in terms of the efficiency of technology in improving one's task performance has a direct effect on continuance intention (Bhattacherjee, 2001a,b; Gefen et al., 2003). The customers would continue to use the technology if they perceive it to provide benefits in performing certain activities and derive satisfaction from service usage (Venkatesh et al., 2003, 2012; Hoehle et al., 2012; Khayer and Bao, 2019; Chen et al., 2009; Rahi et al., 2021; Puriwat and Tripopsakul, 2021; Susanto et al., 2016; Celik, 2008; Patel and Patel, 2018). Because technology-intensive services have no physical presence, any system breakdown or functional difficulties, and the resulting failure to offer services would defy past expectations, leading to discontent and sporadic use (Zhang et al., 2018). Therefore, we propose a positive relationship between PU and satisfaction (H4) and CI (H5).

### 2.4 Cognitive acceptance: perceived trust

Any effort to foster relationships has to be centered on creating trust in uncertain virtual environments (Chaudhuri and Holbrook, 2001). Brand trust is measured by the reliability and honesty of a company in fulfilling its obligations to customers availing services in virtual environments where psychological distance and lack of workable rules deter reliable behaviour (Morgan and Hunt, 1994; Mukherjee and Nath, 2003; Cheng et al., 2017). Perceived trust (PT) denoting the expectation that firms would keep promises and provide services with integrity and ensure confidentiality of customer data and their transactions on technology platforms influences mobile services acceptance (Lu et al., 2011) and continuance intention (Kumar et al., 2018; Zhou, 2011a; Chawla and Joshi, 2019). When users trust firms as their partners, they would cognitively accept the value proposition and expend time, and effort, and use various resources to engage positively with the firm. Also, in a risky virtual environment, the perceived risk of transactions can be reduced by building trust which also improves co-creation interactions (Zeithaml et al., 1996). Hence, consumers’ willingness to co-create largely depends on the expectation of a positive outcome in an interaction (Jaworski and Kohli, 2006). Few studies have documented satisfaction, loyalty, and purchase intention as consequences of trust (Wang et al., 2015). Therefore, a direct positive effect on trust in FinTech payments on willingness to co-create services (H6), satisfaction (H7), and CI (H8) is expected.

### 2.5 Normative acceptance: social influence

The susceptibility to interpersonal influence and group norms causes internalization of obligations, the judgment of value offerings, and willingness to comply with requests (Sunshine and Tyler, 2003; Burger, 1999). Compliance, identification, and internalization are examples of social influence (SI), in which an individual accepts influences to earn either extrinsic or intrinsic benefits that are consistent with their value systems, or to maintain a satisfactory self-defining relationship with a group (Goodwin, 1987; Kelman, 1961). Consumers evaluate products and services by their contribution to the enhancement of social values such as social approval, and status (Kim et al., 2013). Consumer preferences are more likely to be determined when social standards are effectively enforced, meaning that participation in the co-creation of services under social influence is more likely. When social norms are effectively enforced, consumers’ participation in the co-creation of services is more likely (Cialdini et al., 1999; Sutinen and Kuperan, 1999). When people place a higher value on interpersonal information than private information, word-of-mouth information from family, friends, and others becomes critical in CI (Godes and Mayzlin, 2004). SI is a powerful predictor of customer involvement and participation in virtual communities, as well as purchasing behaviour (Stibe et al., 2013; Song and Kim, 2006), Dholakia et al. (2004). Several scholars investigating the effect of SI on financial technology adoption and continuous usage have proved a positive association (Chen et al., 2009, 2012; Bhattacherjee and Lin, 2015). Therefore, we hypothesize a positive relationship between social influence and willingness to co-create services (H9) and CI (H10).

### 2.6 Behavioural acceptance: willingness to co-create services

**Figure 1:**
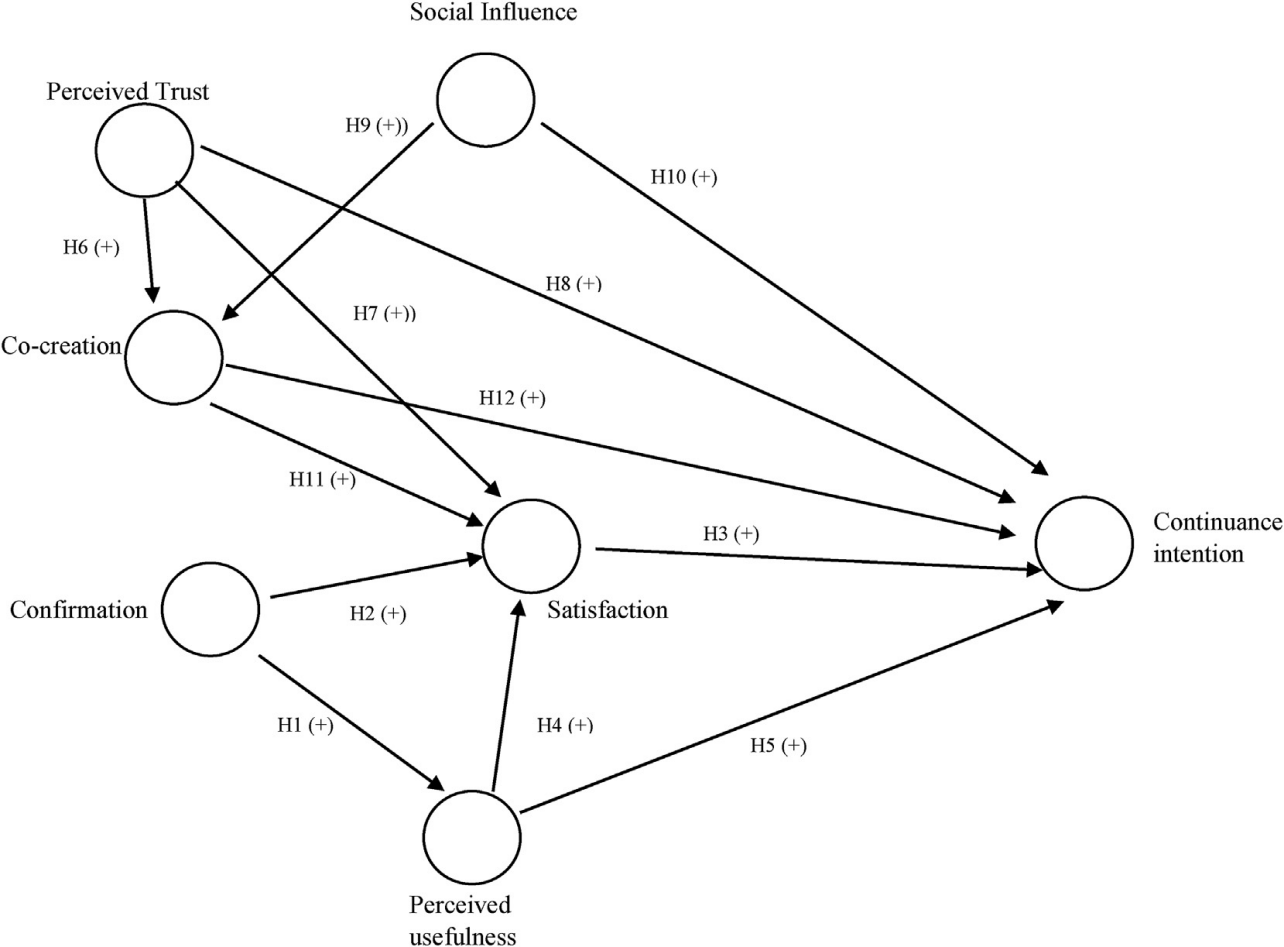
Conceptual framework.

Customers' roles have shifted from passive receivers of services in a transactional connection to active players in deciding service experience. Co-creation (CCR) creates economic value for the customers (Chan et al., 2010) by providing an opportunity to participate in the design of customized products/delivery of services and acquire better knowledge and control over the outcome of services (Auh et al., 2007). According to the service-dominant logic, customers seek solutions to their problems and in interactions between users and service providers, generate value jointly and reciprocally for themselves and the organization by integrating resources and capabilities (Vargo et al., 2008; Vargo and Lusch, 2008; Lusch et al., 2007; Auh et al., 2007). Direct interactions between a service provider's value proposition of its resources and capabilities (operant and operand) and consumers' active engagement in co-creating experiences lead to mutual resource integration and collaboration targeted at mutually beneficial outcomes such as customer satisfaction and CI (Skålén et al., 2015; Shulga and Busser, 2020; Vargo et al., 2008; Jaworski and Kohli, 2006; Zeithaml et al., 1996). Customers who accept the value proposition are more likely to participate in service co-production and report better levels of satisfaction, which leads to repeat purchases (Vargo and Lusch, 2004; García-Haro et al., 2015) and continuous usage (Islam et al., 2019; Kaur et al., 2012; Vargo and Lusch, 2004). Therefore, we hypothesize a direct relationship between willingness to co-create services and satisfaction (H11) and CI (H12). The scope of co-creation in this paper is restricted to activities and interactions between the customers-to-firm (Figure 1).

## 3 Methodology

### 3.1 Measurement tools

A cross-sectional descriptive study using a validated questionnaire measuring several constructs that affect continuance intention was carried out in India. Even though the current study uses a cross-sectional technique to analyze users' intentions to continue using FinTech payment services rather than actual use, earlier research has validated the current study technique (Bhattacherjee, 2001a,b; Hellier et al., 2003). A comprehensive multi-item scale was adapted to measure CF and ST (Bhattacherjee, 2001a,b; Bhattacherjee and Premkumar, 2004), PT (Venkatesh et al., 2011; Suh and Han, 2002), co-creation (Elsharnouby and Mahrous, 2015), PU(Venkatesh et al., 2011; Bhattacherjee and Premkumar, 2004), SI (Taylor and Todd, 1995), and continuance intention (Bhattacherjee, 2001a,b). The variables were measured using a five-point Likert scale with 1-highly disagree and 5-higher agree to measure the agreeability to various items on the scale. A pilot study was carried out involving 46 respondents to assess the reliability and validity of the survey tool. The common method bias was checked using the unmeasured marker variable technique (Lindell and Whitney, 2001). In partial least squares (PLS) analysis, the unmeasured marker variable was inserted, and the change in R^2^ of the target construct (CI) was 7.8%, which is less than 10%. As a result, there was no common method bias in the data set.

### 3.2 Sampling and data collection

The study is based on the primary data collected from the users of payment services offered by banks and FinTech companies. The absence of data on the population using FinTech payment services and the inaccessible sampling frame of FinTech customers motivated us to collect data using online methods. Many scholars have previously employed this method because of its benefits which include increased access to service users, less social desirability bias, addressing hard-to-reach groups and eliciting honest responses from respondents (Kalinić and Marinković, 2020; Zhou, 2011b; Llieva et al., 2002; Wright, 2005; Darmansyah et al., 2021; Shree et al., 2020). In the first phase of the study, we could gather around 225 user emails from the selected big banks and FinTech companies in August 2020. In the second phase, we asked the respondents who took part in the survey to share the email address of their acquaintances and we sent electronic emails during October and November 2020. We could receive responses from 478 respondents with complete information and 15 were incomplete responses. A total of 463 questionnaires were used for further analysis. The sample had a larger proportion (53.2%) of users in the younger age group (below 30 years) followed by those between 30-40 years (34.6%), one-fifth of respondents had bachelor's degrees and an annual income of 34.1% of respondents was INR 1,00,000. The majority of respondents used FinTech services for paying for essentials (48%), utility bills (mobile recharge, electricity, rent) (32.7%), online purchases (4.7%), and all of these (35.7%).

### 3.3 Estimation procedure

The PLS method was used since it does not assume normality assumptions, and is suitable for large and small samples. The hypotheses were tested using the following steps, (a) multicollinearity was calculated to see for any correlation greater than 0.9 between observed variables, (b) assessment of measurement models for reliability (internal consistency reliability) and validity (convergent and discriminate validity), and (c) evaluation of the structural model to test hypothesized relationships by obtaining the path coefficients (Hair et al., 2017). Model fit was assessed to know the significance and strength of relationships, implications of the coefficient of determination, and whether the estimated model fits the observed data. Effect size f^2^ is used to evaluate how much the endogenous construct contributed to the R^2^ values. When an exogenous construct is first included in the model (R^2^ included) and then subsequently deleted (R^2^ excluded), it changes the R^2^ value of the endogenous construct. Q^2^ values, which were determined using the blindfolding process to obtain cross-validated redundancy measures for each endogenous construct were used to assess the predictive relevance of the model. These values should be greater than zero. The omission distance of seven (D = 7) implies that every seventh data point of the target construct's indicators are eliminated in a single blindfolding round. Since the blindfolding procedure has to omit and predict every data point of the indicators used in the measurement model of a certain latent variable, it comprises seven blindfolding rounds. The guidelines provided by Hair et al. (2017) were followed to do the blindfolding procedure and the maximum number of iterations was 400. The effect size q^2^ measures the relative predictive relevance of exogenous constructs for an endogenous construct. It is used to assess the changes in Q^2^ values in the path model when the construct was initially included (Q^2^ included) and thereafter, estimated without it (Q^2^ excluded). The values for the small, medium, and large effects are 0.02, 0.15, and 0.35 respectively (Hair et al., 2017).

## 4 Results

### 4.1 Measurement model assessment: reliability

We measured reliability indicators such as composite reliability (construct reliability) and outer loadings of the indicators of all constructs' values exceeded the threshold of 0.70 (Table 1).

**Table 1:** Reliability of items and constructs.

Construct	Indicators	Outer loadings	Composite reliability	AVE
Willingness to co-create	CCR1 Give feedback about the company's P2P services/products	0.737	0.894	0.627
	CCR2 Discuss needs and wants related to the company's P2P services/products	0.797		
	CCR3 Engage in communications with other customers about the company's P2P services/products	0.813		
	CCR4 Suggest improvements to current P2P products and services	0.815		
	CCR5 Suggest new P2P products and services	0.794		
Social influence	SI1: My friends/colleagues frequently use P2P payment services.	0.814	0.886	0.609
	SI2: The people who are close to me would agree with me using P2P payment system	0.820		
	SI3: My relatives/family members frequently use P2P payment services.	0.712		
	SI4: The people whose opinions I value would approve of me using P2P payment system	0.810		
	SI5: I think a good number of people use P2P payment services during the pandemic.	0.754		
Continuance intention	CI1: I would continue to see myself using fintech P2P payment apps for handling my payment needs even after the pandemic.	0.904	0.920	0.793
	CI2: I predict that I will use fintech P2P payment services in the future.	0.909		
	CI3: I plan to use fintech P2P services for all my needs.	0.856		
Confirmation	CF1: My experience with using fintech P2P payment app was better than what I expected.	0.899	0.903	0.823
	CF2: The level of service provided by fintech companies was more than what I expected	0.915		
	CF3: Overall, most of my expectations from using fintech P2P payment app were confirmed	0.910		
Satisfaction	ST1: I am satisfied with the fintech P2P payment usage.	0.812	0.858	0.751
	ST2: I think I did the right thing by deciding to use fitnech P2P payment services	0.857		
	ST3: Overall, I am delighted with fintech P2P payment app usage.	0.918		
Post-usage Usefulness	PU1: Using fintech P2P services makes it easier for me to conduct transactions	0.876	0.913	0.723
	PU2: Using the fintech P2P services enhances the effectiveness of my payment activities/services	0.876		
	PU3: Using fitnech P2P services would improve the quality of payment transactions performed.	0.818		
	PU4: Using fitnech P2P apps would make it easier to access payment services.	0.829		
Perceived trust	PT1: I trust the transactions done by using fintech P2P app.	0.927	0.933	0.874
	PT2: I think that this fitnech firm offers genuine services in keeping its promise.	0.943		
	PT3: This fintech firm provides P2P payment services in my best interest.	0.915		

Source: primary survey.

### 4.2 Convergent and discriminant validity

All constructs had an average variance extracted (AVE) value of more than 0.5 indicating convergent validity (Table 1). The Fornell-Larcker Criterion as shown in Table 2a establishes discriminant validity since the square root of the AVE of each reflective construct is higher than its highest correlation with any other construct. Heterotrait-Monotrait Ratio (HTMT), which measures discriminant validity, was less than 0.9 (Table 2b). The results of the multi-collinearity test are shown in Table 3, variance inflation factor (VIF) value was less than 5 which indicates no issue with collinearity.

**Table 2:** Discriminant validity.

a. Fornell-Larcker Criterion							
	CI	CCR	PT	PU	SI	CF	ST
CI	0.890						
CCR	0.236	0.793					
PT	0.583	0.413	0.935				
PU	0.765	0.265	0.506	0.850			
SI	0.687	0.338	0.455	0.757	0.811		
CF	0.661	0.255	0.459	0.792	0.712	0.843	
ST	0.592	0.319	0.565	0.595	0.544	0.619	0.766

	b. HTMT Ratio					
	CCR	CI	CF	PT	PU	ST
CI	0.250					
CF	0.255	0.772				
PT	0.443	0.674	0.516			
PU	0.293	0.877	0.708	0.583		
ST	0.276	0.684	0.606	0.507	0.731	
SI	0.395	0.788	0.840	0.570	0.848	0.737

Source: Primary Survey.

Note: CI: Continuance intention, CF: Confirmation, PT: Perceived trust, PU: Perceived usefulness, ST: Satisfaction, CCR: Co-creation, SI: Social influence.

**Table 3:** Collinearity statistics.

	CI	CCR	PU	ST
CI				
CCR	1.262			1.214
PT	1.709	1.261		1.527
PU	2.732			2.888
SI	2.514	1.261		
CF			1.000	2.725
ST	1.854			

Note: CI: Continuance intention, CF: Confirmation, PT: Perceived trust, PU: Perceived usefulness, ST: Satisfaction, CCR: Co-creation, SI: Social influence.

### 4.3 Structural model: estimating the continuance intention model

**Figure 2:**
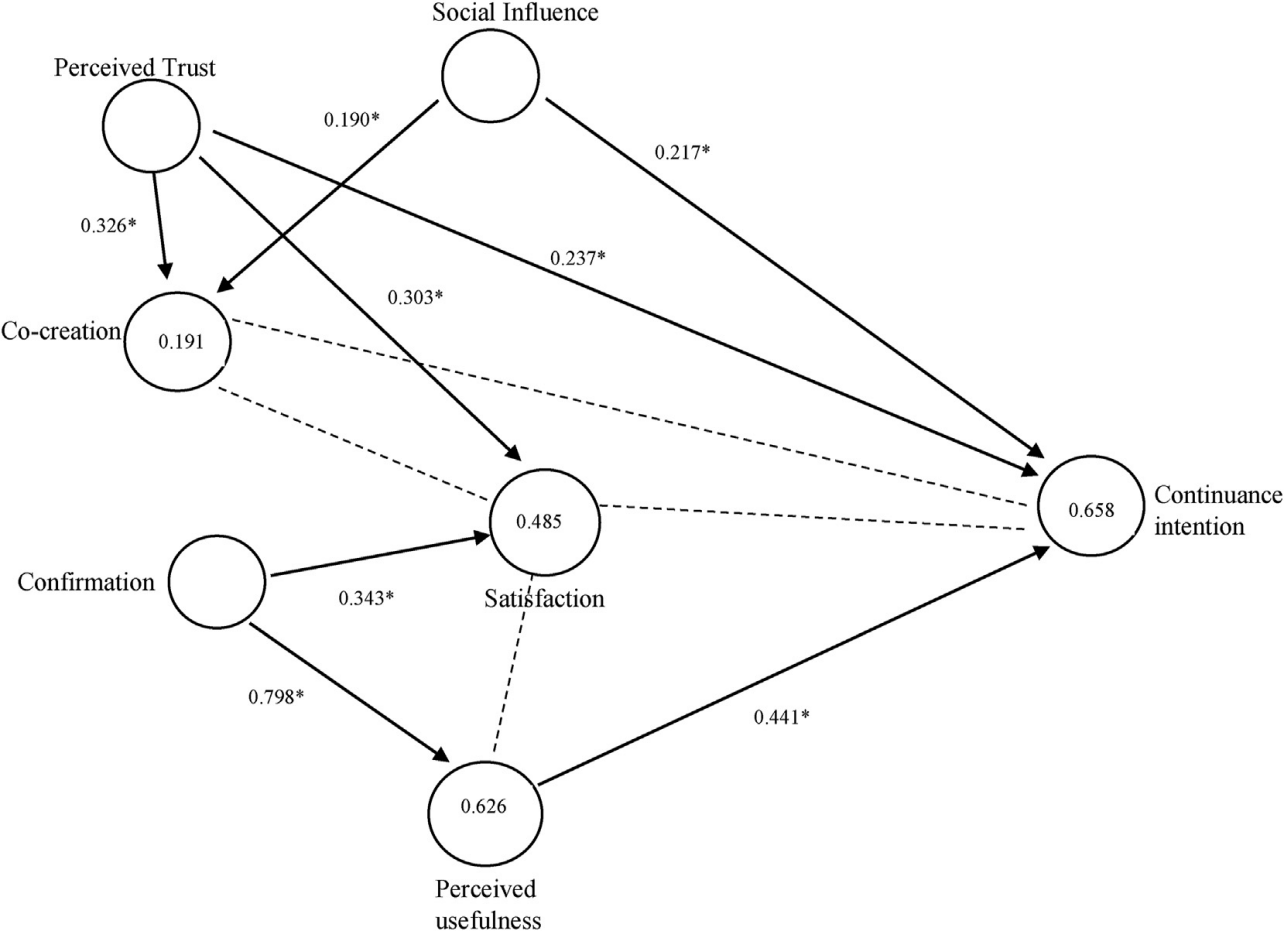
Structural model predicting continuance usage intention: direct effects. ------- denotes non-significant relationship. *∗p < 0.00, ∗∗p < 0.05*.

The five exogenous constructs namely CCR, PT, SI, PU, and ST were assumed to positively influence CI. There were few mediating relationships, ST as a mediator between CCR, PT, PU, and CI, and CCR as a mediator between PT, SI, and CI (Table 4). To assess the significance of the path coefficients, a bootstrapping procedure by selecting the option of ‘no sign changes’ with 2000 samples was carried out (Table 4). R^2^of CI was moderately high at 0.658 suggesting that the structural model has good predictive validity. Similarly, R^2^ of CCR (0.191), PU (0.626), and ST (0.485) are given in Figure 2 (see Figure 1).

**Table 4:** Regression results: Direct, special indirect, and total effects.

Relationship Tested		β	t test value	p value	Decision at 5% level of significance
Direct effects					
H1	CF → PU	0.792	19.228	0.000	Supported
H2	CF → ST	0.343	3.203	0.000	Supported
H3	ST → CI	0.105	1.825	0.069	Not supported
H4	PU → ST	0.152	1.688	0.092	Not supported
H5	PU → CI	0.441	5.064	0.000	Supported
H6	PT → CCR	0.326	4.958	0.000	Supported
H7	PT → ST	0.303	3.956	0.000	Supported
H8	PT → CI	0.237	3.743	0.000	Supported
H9	SI → CCR	0.190	2.382	0.018	Supported
H10	SI → CI	0.217	2.583	0.010	Supported
H11	CCR → ST	0.066	1.305	0.192	Not supported
H12	CCR → CI	−0.085	1.728	0.085	Not supported
Specific Indirect effects					
CF → PU → CI	0.349	4.716	0.000	Supported	
Total effects					
CCR → CI	−0.078	1.583	0.114	Not supported	
CCR → ST	0.066	1.305	0.192	Not supported	
PT → CI	0.243	3.842	0.000	Supported	
PT → CCR	0.326	4.958	0.000	Supported	
PT → ST	0.325	4.396	0.000	Supported	
PU → ST	0.152	5.300	0.000	Supported	
PU → CI	0.457	1.688	0.092	Not supported	
ST → CI	0.105	1.825	0.069	Not supported	
CF → PU	0.792	19.228	0.000	Supported	
CF → ST	0.463	5.877	0.000	Supported	
CF → CI	0.398	5.276	0.000	Supported	
SI → CCR	0.190	2.382	0.018	Supported	
SI → ST	0.013	1.007	0.314	Not supported	
SI → CI	0.202	2.431	0.015	Supported	

Source: primary survey.

Note: CI: Continuance intention, CF: Confirmation, PEOU: Perceived ease of use, PT: Perceived trust, PU: Perceived usefulness, ST: Satisfaction, CCR: Co-creation, SI: Social influence.

The significant path coefficients implying a strong positive relationship between CI and PU (β 0.441, p < 0.00), PT (β = 0.237, p < 0.00), and SI (β = 0.217, p < 0.05) has been observed (Table 4). The specific indirect effect was observed along the paths, CF → PU → CI (β = 0.349, p < 0.05). The path coefficients for CCR → CI, CCR → ST, PU → ST, and ST → CI (0.075) were not significant. All null hypotheses except H3, H4, H11, and H12 were rejected. No mediation effect of CCR (SI → CCR → CI, PT → CCR → CI), and ST (CCR → ST → CI, PU → ST → CI, PT → ST → CI) was found. The model had an SRMR value of 0.088 which is less than the accepted level of 0.1 and NFI (normed fit index) is closer to 1 (0.729). The significant Chi-square test and rms_theta values of 0.092 indicate a good fit.

The blindfolding procedure was carried out to know the predictive relevance. The model has a Q^2^ value of 0.496 (CI), 0.114 (CCR), and 0.271 (ST) which are larger than zero. The f^2^ analysis (Table 5) reveals that paths CF has a larger effect on PU (1.687) and PU → CI (0.213) and PT → ST (0.119) have a medium effect. PT → CCR (0.105), PT → CI (0.099), SI → CCR (0.036), CF → ST (0.085) and SI → CI (0.056) have a small effect.

**Table 5:** Effect size, predictive relevance, and q2 effect size.

Exogenous variable	Endogenous variable	f2	Q2 included	Q2 excluded	q2 effect size
ST	CI	0.018 (NE)	0.496	0.509	−0.025 (NE)
PU	CI	0.213 (ME)	0.496	0.453	0.085 (SE)
PT	CI	0.099 (SE)	0.496	0.489	0.013 (NE)
CCR	CI	0.017 (NE)	0.496	0.509	−0.025 (NE)
SI	CI	0.056 (SE)	0.496	0.497	−0.001 (NE)
CF	ST	0.085 (SE)	0.271	0.243	0.666 (HE)
PU	ST	0.016 (NE)	0.271	0.271	0.000 (NE)
PT	ST	0.119 (ME)	0.271	0.242	0.039 (SE)
CCR	ST	0.007 (NE)	0.271	0.273	−0.002 (NE)
SI	CCR	0.036 (SE)	0.114	0.098	0.018 (NE)
PT	CCR	0.105 (ME)	0.114	0.063	0.057 (SE)

Source: Primary survey.

Note: CI: Continuance intention, CF: Confirmation, PEOU: Perceived ease of use, PT: Perceived trust, PU: Perceived usefulness, ST: Satisfaction, CCR: Co-creation, SI: Social influence NE: no effect; SE: small effect HE: high effect.

### 4.4 Importance-performance map analysis

**Figure 3:**
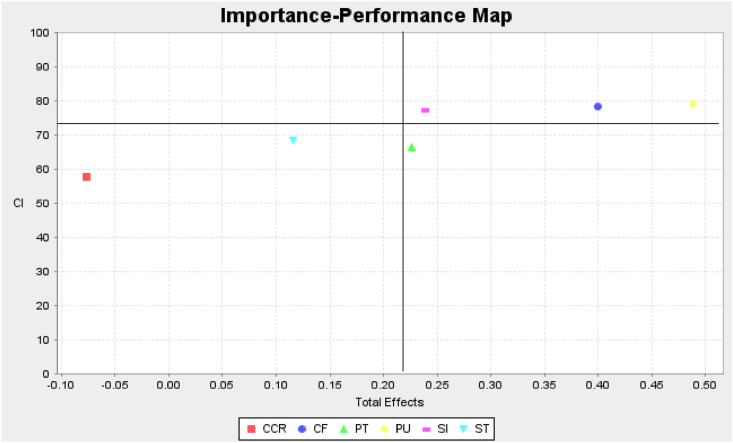
Important-performance map analysis: continuance usage intention. Note: CI: Continuance intention, CF: Confirmation, PT: Perceived Trust, PU: Perceived usefulness, ST: Satisfaction, CCR: Co-creation, SI: Social Influence.

The results show that PU has relatively higher importance (0.457) on CI and greater performance (79.06) followed by CF (0.398 and 78.28 respectively), and SI (0.202 and 77.37 respectively) but ST has low importance (0.105) and performance (68.48). However, urgent managerial actions are needed to improve trust since it has a higher effect but lower performance in influencing CI (0.243 and 66.46 respectively) (Figure 3 and Table 6).

**Table 6:** IPMA analysis-total effects and performance for continuance usage intention.

Constructs	Total effects	Performance
Co-creation	−0.078	57.65
Confirmation	0.398	78.28
Perceived trust	0.243	66.46
Perceived usefulness	0.457	79.06
Social Influence	0.202	77.37
Satisfaction	0.105	68.48

## 5 Discussion

The limited empirical evidence on the influence of customers' acceptance of value proposition in the context of FinTech P2P payment services and its effect on CI prompted us to incorporate the acceptance model into the well-known ECM. The Theory of Acceptance has been mainly used in many scientific and philosophical explorations that provide a theoretical framework to understand customers' acceptance of value propositions. The present paper concludes the positive influence of cognitive (trust) and normative (social influence) acceptance on behavioural acceptance (willingness to co-create) and continuance usage intentions. Value acceptance is more likely if companies build trust, persuade ‘significant others,' and deliver on the promises made in the value proposition. Also, we found continuance intention to use FinTech P2P payments to be supported by ECM constructs namely PU and CF. The confirmation has a positive influence on ST and PU. Moreover, PU fully mediates the relationship between CF and CI. Path analysis reveals that PU has a large effect on CI, followed by CF, PT, and SI. Therefore, ECM does certainly explain the majority of variance in endogenous construct, whereas cognitive and normative acceptance of value proposition not only influence behavioural acceptance but also contribute to continuance intention. IPMA suggests the highest total effect and greater performance of PU on CI, followed by CF and SI. PT has higher importance but lower performance.

Following the findings of the present study (H1 and H2), few scholars have documented the crucial role of CF on PU and ST (Bhattacherjee, 2001a,b; Bhattacherjee and Premkumar, 2004). IPMA results also support a higher effect (0.398) and performance (78.28) of CF on CI. Prior expectations are usually based on mass media (including social media) and publicly available information such as industry reports. The relative influence of these elements, rather than personal experience, tends to make the formulation of expectations unpredictable (Bhattacherjee and Barfar, 2011). On the contrary, post-adoption expectations about PU often would be stronger, more enduring, and predictable owing to the first-hand experience. Confirmation of prior expectations, therefore, increases post-purchase PU and satisfaction and decreases the possibility of switching to competitors by positively influencing continuous intention to use FinTech services. Thus, confirmation of initial expectation from the actual use and fulfillment of earlier perceived usefulness has a significant effect on satisfaction and post-adoption PU.

As an overall post-adoption evaluation, satisfaction was not found to be one of the determinants of CI (H3). It does not mediate the effect of confirmation, PU, and PT on CI. Although in marketing research, consumers’ continuance behaviour depends on satisfaction, not only as a direct effect (Bhattacherjee, 2001a, Bhattacherjee, 2001b) but also as a mediator effect (Oliver, 1980), the present study could not support the findings of other studies (Zhang et al., 2011; Rose et al., 2012; Oghuma et al., 2016; Bhattacherjee, 2001a,b).

Post-usage usefulness affects CI (H4) when initial usage experiences with the application would strengthen positive perceptions about performance such as convenience, saving of time and money, and improved efficiency. Several studies support that PU in terms of convenience of payments, speed of transactions on virtual platforms, and service effectiveness benefit the consumers (Zhou et al., 2010; Venkatesh et al., 2003; Bhattacherjee, 2001a,b). Post-purchase PU would be higher if the firm continues to provide streamlined and convenient means of payment transactions. When the consumers become familiar with the FinTech P2P offering, the likelihood of appreciating the benefits of new financial technology and its continuous usage increases because of the clarity, and capability to use the new digital payment system.

The present study also observed a direct effect of perceived trust on co-creation (H6) and satisfaction (H7). Similarly, a greater effect but lower performance of PT on CI (H8) was seen. In developing countries like India where the internet and information technology infrastructure and regulatory framework and policies are evolving, trust is crucial to retain customers and establish long-lasting relationships with firms. Trust in online payment systems and consumer involvement has been suggested to improve the continuance intention of technology use (Venkatesh et al., 2011; Zhou, 2011a; Lu et al., 2011). User interactions with service providers necessitate sharing of ideas, service reviews, and grievances with companies, as well as giving information to the electronic medium such as recommendations and reviews, over which enterprises rarely exert control (Novak et al., 2000). In the absence of physical contact, such communications necessitate trusting relationships where users would be more willing to share information with service providers and anticipate fruitful future interactions (Johnson and Grayson, 2005; Chai and Kim, 2010). When an individual does not trust a party or a company, the likelihood of sharing information or interaction would be lower since he or she might expect a negative outcome from such interactions (Bharti et al., 2014; Chepurna and Criado, 2018). Increased trust in FinTech P2P payment firms would result in more interactions, giving rise to higher satisfaction and repeat transactions. Consumers would be willing to share information and actively participate in co-production or co-designing products/services only if they trust the service providers. In a trusting environment, consumers perceive that their ideas or suggestions would have beneficial effects when other customers and service providers cooperatively engage and assist in building reputation and creating value for all the parties concerned. Therefore, if firms provide opportunities for engaging with companies in trusted value-creating interactions, users would be more satisfied and continue to use P2P services.

Social influence directly affects CCR (H9) and CI (H10). Indian customers tend to rely heavily on the advice given by ‘referents’ or ‘significant others’ in making continuance use of P2P payment services. The current study also reveals that consumers with a greater level of SI are more likely to engage in co-creation. Few studies confirm our findings that SI improves the perception of relative advantages leading to active involvement in value-creation activities (Kim et al., 2013). Edvardsson et al. (2011) emphasized the mechanism of social exchange and value co-creation by focusing on social dominant logic. Individuals continuously use services to gain social value measured as approval by social influencers, self-identification, social acceptance, and self-esteem. Also, customers' participation in virtual communities is influenced by their identification and internalization (Bagozzi and Dholakia, 2002). Internalization, an informational influence, motivates an individual to incorporate a referent's beliefs and recommendations into one's own belief as evidence for reality. Compliance can impact users' propensity to participate in co-creation and continuance use since customers expect rewarding service experiences such as need satisfaction or price discounts, gifts, and so on.

The study suffers from a few limitations as reported in other studies on ECM and acceptance theory. Although several factors influencing continuance intention were incorporated in the model, the role of culture and other social factors, government support, regulatory factors, and cognitive factors were not studied. In addition, the effect of acceptance of value proposition on the well-being of users and organizations can also be studied. Future researchers could study a modified ECM model in the context of peer-to-peer lending, InsureTech, and Robo-advisory services. The application of projective techniques, random sampling techniques, and longitudinal studies may strengthen the findings. This paper focuses on demand-side factors, hence future studies could explore the supply-side factors such as access to the internet and institutional and regulatory on the continued use of FinTech P2P payment services. The present study did not include demographic and regional variables which future studies could include to explain CI to use the FinTech P2P payment services. Because of COVID-19 lockdowns and restrictions on physical movements, the random sampling method could not be applied for sample selection, hence the findings of the study need to be interpreted judiciously.

### 5.1 Managerial implications

Because continuance intention is determined by trust (cognitive acceptance) and social influence (normative acceptance), FinTech firms must showcase the reliability and credibility of their service on the firm's website and incorporate it into marketing materials. In comparison to other service providers, it must also give a unique co-created experience and inspire social influencers to propagate positive word-of-mouth. Condensing these views into a brief value proposition might help companies persuade people to adopt their value proposition and turn “one-time” users into long-term customers. Therefore, companies must present a coherent, consistent, and compelling message to the customers about the value they could expect from them, and this message must be delivered holistically across the customer acquisition and retention process.

The confirmation of expectations influences PU and satisfaction through the process of reasoned action and indirectly influences CI. The most common benefits of FinTech are extrinsic motivational factors that are manifested in the achievement of specific goals such as financial benefits (cost reduction, financial rewards), the flexibility of transactions (mobility, seamless access), and convenience. If FinTech firms' offerings meet or exceed consumers’ prior expectations, it can enhance the belief in the ability of firms to meet future expected benefits and satisfy future needs. Although FinTech companies are transforming payment services and rewriting the rules of the game by providing innovative and desired financial products and services, they can improve PU by improving customer productivity, transparency, and transaction speediness when compared to traditional banking and other digital channels.

The positive belief built on favourable outcomes during the recurrent interactions strengthens the exchange relationship and ensures that consumers get anticipated results and service satisfaction. A direct effect of confirmation and co-creation on satisfaction indicates a greater role of FinTech firms in mobilizing internal resources to understand customer's desires and build user-friendly and reliable platforms for personalized experiences, provide reliable and relevant information for a meaningful experience, and access to resources that facilitate fruitful dialogues and gauge customers' expectations. Moreover, FinTech companies should strategically employ advanced analytics, collective intelligence, and machine learning to address unique expectations and redress grievances. If the design and layout are user-friendly and prioritize consumers' needs by providing virtual (technical and messaging such as SMS, WhatsApp, and live chat) support, FinTech might boost perceived usefulness.

Trust is a prerequisite for customers to accept new technology-based platforms like FinTech for conducting financial transactions. In virtual non-face-to-face platforms involving significant risks, ongoing trust is necessary to provide correct information on the value proposition and alleviate anxiety associated with complex transactions in an internet environment where unfair and fraudulent practices, theft of personal data, and misrepresentation of information are rampant. So, FinTech companies must invest resources in establishing trust in their services by providing third-party reviews, transparency of information on products and services, protecting sensitive personal information short videos on the use and benefits of financial tools, and safe payment methods, and displaying security seals or accreditation on the website or mobile applications. These strategies would not only enhance users' co-creation activities but also encourage continuous use of FinTech payment systems.

Customers will be more ready to participate in service co-creation if they believe information offered by ‘significant others’ on value offerings (benefits, satisfaction of functional demands, or self-expression needs) meets their expectations. Similarly, the involvement in co-creation would be stronger if the customers socially identify themselves and find their participation intrinsically rewarding. To leverage their influence on consumers, businesses could identify ‘significant others’ and influence them through social media and digital marketing tools.

## 6 Conclusion

During the COVID-19 outbreak, the FinTech industry has witnessed substantial growth by providing convenient, flexible, and efficient financial transactions without time or geographical barriers. Customers' continued use of FinTech P2P payments hinges on confirmation of expected benefits, post-adoption usefulness, social influence, and trust. The firm's offering should consistently match or surpass users' expectations by providing speedy and reliable transactions without any system errors or service breakdown and by improving service performance When a firm proactively incorporates customer expectations in its offerings and deploys resources to fulfill its promises, confirmation of initial expectations would increase post-usage expected benefits and satisfaction and thereby influence continuance usage intentions. The cognitive acceptance can be improved by consistently demonstrating reliability and honesty and building a trusting environment that encourages users to innovate and co-create for value-embedded service delivery. In addition to building trust by ensuring privacy, transparency, and security of transactions, the managers should capitalize on social influence towards providing a platform for co-creation where the customers could access information related to products, develop networks, and communicate their ideas, preferences, and experiences on social media and web conversations.

## Declarations

### Author contribution statement

1 Conceived and designed the experiments: Basri Savitha and Iqbal Thonse Hawaldar.
2 Performed the experiments (collection of data): Basri Savitha, Iqbal Thonse Hawaldar, and Naveen Kumar K.
3 Analyzed and interpreted the data: Basri Savitha and Iqbal Thonse Hawaldar.
4 Contributed reagents, materials, analysis tools or data: Basri Savitha, Iqbal Thonse, Hawaldar, and Naveen Kumar K.
5 Wrote the paper: Basri Savitha, Iqbal Thonse Hawaldar, and Naveen Kumar K.

### Funding statement

This research did not receive any specific grant from funding agencies in the public, commercial, or not-for-profit sectors.

### Data availability statement

Data will be made available on request.

### Declaration of interest's statement

The authors declare no conflict of interest.

### Additional information

No additional information is available for this paper.
